# Different coding characteristics between flight and freezing in dorsal periaqueductal gray of mice during exposure to innate threats

**DOI:** 10.1002/ame2.12276

**Published:** 2022-10-12

**Authors:** Denghui Liu, Shouhao Li, Liqing Ren, Xinyu Liu, Xiaoyuan Li, Zhenlong Wang

**Affiliations:** ^1^ School of Electrical and Information Engineering Zhengzhou University Zhengzhou China; ^2^ School of Intelligent Manufacturing Huanghuai University Zhumadian China; ^3^ School of Life Sciences Zhengzhou University Zhengzhou China

**Keywords:** C57BL/6 mice, dorsal periaqueductal gray, flight and freezing, innate threats, neural coding

## Abstract

**Background:**

Flight and freezing are two vital defensive behaviors that mice display to avoid natural enemies. When they are exposed to innate threats, visual cues are processed and transmitted by the visual system into the emotional nuclei and finally transmitted to the periaqueductal gray (PAG) to induce defensive behaviors. However, how the dorsal PAG (dPAG) encodes the two defensive behaviors is unclear.

**Methods:**

Multi‐array electrodes were implanted in the dPAG nuclei of C57BL/6 mice. Two kinds of visual stimuli (looming and sweeping) were used to induce defensive behaviors in mice. Neural signals under different defense behaviors were recorded, and the encoding characteristics of the two behaviors were extracted and analyzed from spike firing and frequency oscillations. Finally, synchronization of neural activity during the defense process was analyzed.

**Results:**

The neural activity between flight and freezing behaviors showed different firing patterns, and the differences in the inter‐spike interval distribution were mainly reflected in the 2–10 ms period. The frequency band activities under both defensive behaviors were concentrated in the theta band; the active frequency of flight was ~8 to 10 Hz, whereas that of freezing behavior was ~6 to 8 Hz. The network connection density under both defense behaviors was significantly higher than the period before and after defensive behavior occurred, indicating that there was a high synchronization of neural activity during the defense process.

**Conclusions:**

The dPAG nuclei of mice have different coding features between flight and freezing behaviors; during strong looming stimulation, fast neuro‐instinctive decision making is required while encountering weak sweeping stimulation, and computable planning late behavior is predicted in the early stage. The frequency band activities under both defensive behaviors were concentrated in the theta band. There was a high synchronization of neural activity during the defense process, which may be a key factor triggering different defensive behaviors.

## Graphical abstract

Flight and freezing are two vital defensive behaviors in the process of avoiding natural enemies in mice. We investigated the neural activity in dPAG nuclei of mice under two defensive behaviors, and found different coding features between flight and freezing behaviors.

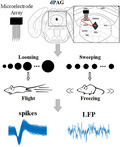

## 1 INTRODUCTION

When animals experience stimuli that arouse their fear or disgust, they demonstrate a strong physiological and psychological stress response accompanied by defensive behaviors.^1^ In particular, rodents typically exhibit appropriate innate defensive behaviors based on the threat's urgency and the environment's complexity,^2^ such as flight, freezing, and defensive aggression.^3^, ^4^ Flight and freezing are the two most vital innate defensive behaviors that mice exhibit to avoid natural enemies,^5^ but how the brain processes and transmits quickly threat information and then generates appropriate defensive responses needs to be further studied.

Researchers have designed many experimental paradigms in the laboratory based on sight, smell, or hearing to induce fear responses in rodents by simulating threats in the natural environment.^6^, ^7^, ^8^, ^9^ In visual stimuli, rapidly expanding black discs (looming) proved to simulate an approaching predator in the sky, which can induce flight behavior in mice.^10^ Another study found that mice tended to choose a flight behavior when they dealt with an approaching predator in the sky, whereas they were more likely to freeze when they faced a hovering predator.^11^ Studies have shown that the subcortical visual pathway plays a vital role during the processing of fear visual information,^12^, ^13^, ^14^ and the periaqueductal gray (PAG) is the last common pathway for multiple defense responses.^15^, ^16^, ^17^, ^18^

The general understanding of the role of the PAG nuclei in defensive behavior is that the dorsal PAG (dPAG) and the ventral PAG (vPAG) are responsible for different behaviors; that is, dPAG activity is primarily associated with flight. In contrast, the neural response of vPAG is more likely to cause freezing^19^, ^20^, ^21^; stimulation of the ventral and dorsal regions of the rat PAG can induce flight and freezing behaviors, respectively.^22^ On the contrary, studies have also shown that, in addition to flight behavior, dPAG can control risk assessment and freezing.^23^, ^24^, ^25^, ^26^ Lesions of the dPAG in rats were found to block the defensive responses to predators, such as flight and freezing.^27^ Optogenetic stimulation of excitatory neurons in dPAG resulted in increased flight and freeze behaviors, suggesting that cell‐type‐specific activation of excitatory neurons in dPAG can mediate both flight and freeze defense responses.^26^ Some researchers also believe that innate‐freezing and learned‐freezing behaviors are controlled by dPAG and vPAG, respectively.^28^ Based on studies of electrical stimulation, injury, and pharmacology,^29^ the PAG matter has been recognized as an essential part of the neural circuits that elicit defensive responses such as flight and freeze in response to threats. However, it is not clear whether dPAG is involved in these two defensive behaviors and how to encode both defense behaviors.

To reveal the neural coding characteristics of dPAG nuclei in regulating flight and freezing defense behaviors, this study used looming and sweeping visual stimuli to induce different innate defense behaviors in C57BL/6 mice. And by implanting micro‐array electrodes in the dPAG nuclei of mice, the neural activity signals under two defensive behaviors were recorded. The coding characteristics of different defensive behaviors analyzed from spike firing and local field potential (LFP) oscillation perspectives will help understand and reveal the coding mechanism of innate defense behaviors.

## 2 MATERIALS AND METHODS

### 2.1 Animal preparation and surgery

Three adult male C57BL/6 mice (weight: 25 ± 3 g, aged: 8–14 weeks) were used in this study. The mice were purchased from Henan Experimental Animal Center and housed individually under the following conditions: 23–25°C, 12–h light–dark cycle (lights on from 8:00 a.m. to 8:00 p.m.), and free access to water and food. The experiments conformed to the Institutional Guidelines for the Care and Use of Laboratory Animals of Zhengzhou University and the National Institutes of Health Guide for Care and Use of Laboratory Animals (GB 14925‐2010). Institutional Animal Care and Use Committee of Zhengzhou University (ZZUIRB2022‐44).

For cranial surgery, the mice were anesthetized with sodium pentobarbital (1%, 80 mg/kg, 0.5%–1.5% isoflurane in medical‐grade oxygen for the remainder of surgery adjusted to keep pain reflexes subthreshold) and then fixed on a stereotaxic apparatus (KOPF940; Kopf Instruments). The body temperature of mice was maintained at 37–38°C using a heating pad. A surgical drill was used to create a 2 × 2 mm cranial opening in the skull. The dura was carefully removed at the insertion site to facilitate the delivery. Two small trepanations (diameter: 2 mm) were drilled over the cerebellum and the olfactory bulb to place silver ball electrodes on top of the dura used to ground (olfactory bulb) the animal and to provide the reference signals for local field potentials (cerebellum). A 16‐channel (4 × 4) microelectrode array (length: 5 mm, pitch: 200 μm, Hong Kong Plexon Inc.)^30^ was inserted through the dura mater into the dPAG nuclei (anterior–posterior: −3.88 mm; medial–lateral: +0.38 mm; dorsal–ventral: −2.3 mm) according to *The Mouse Brain in Stereotaxic Coordinates* (third edition) at an angle perpendicular to the cortical surface. A slight dimpling of up to 100 μm of the cortex was accepted. Increasing amplitude was avoided in all cases by decreasing the speed with which the electrodes penetrated the brain. The space between the dura and the lower part of the microelectrode array was then filled with antibiotic ointment (Nebacetin, Astellas Pharma). After the electrodes were implanted, they were fixed using dental cement. Antibiotic ointment was applied to the wound. The loose skin was sutured and carefully attached to the implant. After surgery, the animals were kept warm, and Baytril was injected into the abdominal cavity of the animals for anti‐inflammatory treatment. The extra load on the animals' heads (∼3 g) did not affect their posture or grooming behavior. One week after surgery, with good recovery, the animals were handled by the experimenter and systematically desensitized to all experimental procedures.

### 2.2 Experimental procedures

**FIGURE 1:**
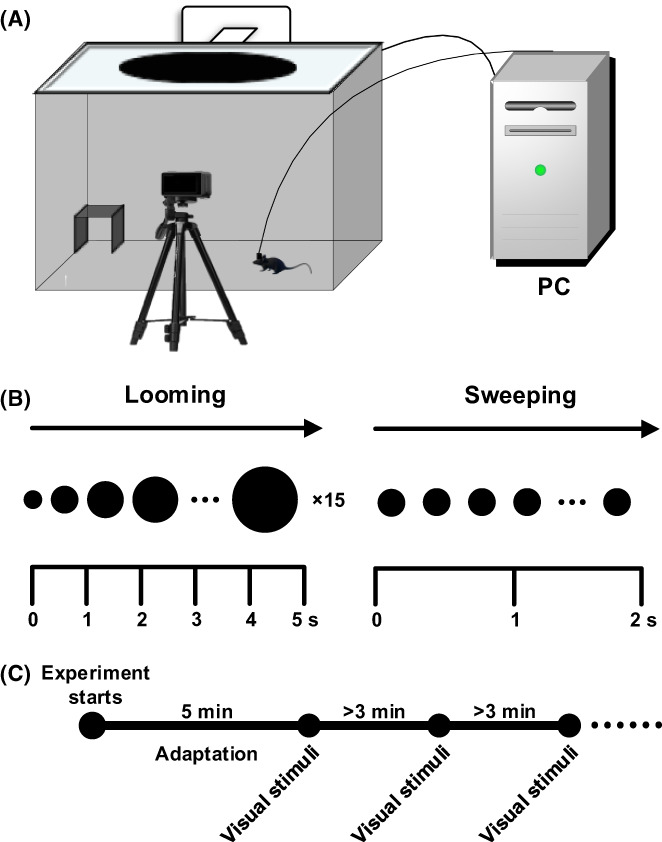
Experimental device and scheme. (A) Experimental device. (B) Two kinds of visual stimuli protocols. (C) Experimental program.

The experimental box was made of acrylic sheets (40 × 40 × 30 cm, width × length × height). The center of the arena was a smaller concentric square, covering 25% of the arena floor area (20 × 20 cm), and an LCD monitor was placed on top of the box. A black nest (10 × 10 × 12 cm) was placed at the inner corner of the box to provide a safe place for the mice during visual stimulation.^10^ Meanwhile, a camera (C920 PRO, 30 fps, Logitech) was placed above the front of the experimental box to record animal behavior (Figure 1A). Visual stimulus programs were written using the MATLAB toolbox Psychtoolbox‐3. A photodiode was placed at a corner of the screen to detect the onset of visual stimulation, which was used to add time marks to neural signal recording to ensure the synchronization of electrophysiological signals with visual stimuli.

This paper uses two visual stimulation protocols (Figure 1B).^11^ One was looming visual stimuli (on a gray background, a black disk rapidly expanded from 2° to 40°, repeated 15 times in 5 s); the other was sweeping visual stimuli (on a gray background, a 5° black disc moved from the left to right of the monitor at 20°/s for 2 s). The day before the experiment, the mice were placed in a chamber, with an LCD screen showing only a gray background, to acclimatize freely for 10 min. During formal investigation, the mice were placed in the experimental box and allowed to move freely for 5 min to adapt, and the baseline data were recorded. Then, when the mice moved to the central area of the practical box, the experimenter manually controlled the keyboard to trigger visual stimuli (the looming and sweeping visual stimuli were presented alternately). The interval of visual stimuli was not less than 3 min, and each mouse was experimented with 5–10 visual stimuli per day (Figure 1C). Before and after each experiment, the experimental chamber was cleaned with 75% alcohol to eliminate odor effects.

### 2.3 Electrophysiological recordings

Electrophysiological signals were recorded using the Cerebus system (Blackrock Microsystems); the electrode impedance of each channel was 0.40–0.60 MΩ, magnified 4000×. The LFP signal was extracted from the raw signal using a 0–250 Hz Butterworth low‐pass filter at a sampling rate of 2 kHz. The spike signal was removed from the raw signal using a 0.25–5 kHz second‐order Butterworth bandpass filter at a sampling rate of 30 kHz. The 50‐Hz power frequency interference and spatial artifact noise in the LFP signal were removed using a least mean square adaptive filter and an adaptive standard average reference filter,^31^ respectively. Spike was extracted from the raw signal using threshold detection and classified using the Skew‐t algorithm.^32^ Units with a precise absolute refractory period in the auto‐correlogram were classified as single units. Signal acquisition was performed while the mice were awake and moving freely.

### 2.4 Data fitting

The one‐phase decay equation was used to fit the latency of the innate defensive behavioral response of mice, and its formula is as follows:

(1) $$Y=\left(Y_{0}-\text{Plateau}\right)\times e^{\left(-K\times X\right)}+\text{Plateau}$$

where *Y*_0_ is the *Y* value when *X* (time) is 0; Platenau is the *Y* value at infinite time; *K* is the rate constant, expressed in reciprocal of the *X* axis time units; *Half‐life* is computed as $\ln\left(2\right)/K$; and *Span* is the difference between *Y*_0_ and Platenau.

The Gaussian distribution equation was used to fit the automatic defensive behavioral response of mice to visual stimuli, and its formula is as follows:

(2) $$Y=\text{Amplitude}\times e^{\frac{{\left(X-\text{Mean}\right)}^{2}}{2\times {\mathrm{SD}}^{2}}}$$

where Amplitude is the height of the center of the distribution in *Y* units, *Mean* is the *X* value at the center of the distribution, and SD is a measure of the width of the distribution.

### 2.5 Spike firing rate

The spike firing rate is the statistical result of the firing activity of the neuron cluster within a specific time window, and the spike firing rate *R*_*i*_ is defined as the ratio of the number of spikes in a given time window to the length of the time window. The *R*_*i*_ is calculated as follows:

(3) $$R_{i}=\frac{S_{i}\left(t_{i}-t_{i-1}\right)}{\mathrm{\Delta t}}$$

where Δt is the length of the time window and $S_{i}\left(t_{i}-t_{i-1}\right)$ is the number of spikes in the time window. In this paper, the length of time window was set to 100 ms.

### 2.6 Continuous wavelet transform

Continuous wavelet transform can adjust the time window size by stretching and translating the mother wavelet, so it has better time and frequency resolution when analyzing neural activity. In this paper, the Morlet wavelet was used as the mother wavelet for time–frequency analysis. The frequency characteristics of neural oscillations were investigated by analyzing the energy change in the LFP signal in dPAG nuclei during flight and freezing. The wavelet transform coefficient ${\mathrm{WT}}_{x}\left(a,b\right)$ at each time point is defined as the convolution of the signal $x\left(t\right)$ with the continuous wavelet $\psi_{a,b}\left(t\right)$:

(4) $$\psi \left(t\right)=\pi^{-1/4}e^{{\mathrm{iw}}_{0}t}e^{-t^{2}/2}$$

(5) $$\psi_{a,b}\left(t\right)={\left|a\right|}^{-\frac{1}{2}}\psi \left(\frac{t-b}{a}\right),\;a,b\in R;a\neq 0$$

(6) $${\mathrm{WT}}_{x}\left(a,b\right)=x\left(t\right)*\psi_{a,b}\left(t\right)={\left|a\right|}^{-1/2}\int x\left(t\right)\psi^{*}\left(\frac{t-b}{a}\right)\mathrm{dt}$$

where $\psi \left(t\right)$ is the mother wavelet, *t* is the time, $\omega_{0}$ is the center frequency, $\psi_{a,b}\left(t\right)$ is the continuous wavelet generated by the mother wavelet, *a* is the scale factor, *b* is the translation factor, and “*” represents the convolution operation. The wavelet transform coefficient *WT* of the LFP signal in the range of 1–80 Hz (equally divided into 256 frequencies) was calculated.

### 2.7 Functional brain network

#### 2.7.1 Synchronization likelihood

When constructing a functional network, measuring the synchronization between the characteristic frequency bands of different channel signals is necessary. The synchronization likelihood (SL) algorithm can not only measure the synchronization between linear and nonlinear signals but also reflect the change in synchronization with time, which is suitable for the synchronization measurement of nonstationary signals such as LFP signals. SL is one of the commonly used functional connectivity indicators. For the detailed calculation process of SL values, see Stam and van Dijk.^33^ The SL value between each channel and other channels constitutes the network connection matrix *G*:

(7) $$G=\left[\begin{array}{cccccc} {\mathrm{SL}}_{1,1} & {\mathrm{SL}}_{1,2} & \cdots & {\mathrm{SL}}_{1,k} & \cdots & {\mathrm{SL}}_{1,n}\\ {\mathrm{SL}}_{2,1} & {\mathrm{SL}}_{2,2} & \cdots & {\mathrm{SL}}_{2,k} & \cdots & {\mathrm{SL}}_{2,n}\\ \vdots & \vdots & \ddots & \vdots & \cdots & \vdots \\ {\mathrm{SL}}_{k,1} & {\mathrm{SL}}_{k,2} & \cdots & {\mathrm{SL}}_{k,k} & \cdots & {\mathrm{SL}}_{k,n}\\ \vdots & \vdots & \vdots & \vdots & \ddots & \vdots \\ {\mathrm{SL}}_{n,1} & {\mathrm{SL}}_{n,2} & \cdots & {\mathrm{SL}}_{n,k} & \cdots & {\mathrm{SL}}_{n,n} \end{array}\right]$$

where *SL*_*k,n*_ = *SL*_*n,k*_, *SL*_*k,n*_ represents the degree of synchronization between channel *k* and channel *n*; the value of SL is between *P*_*ref*_ and 1; *P*_*ref*_ indicates that the signals are not synchronized, and 1 indicates that the signals of the two channels are entirely synchronized. The SL value was calculated using the HERMES toolbox, and the parameter settings were as follows^34^: *d* = 10, *τ* = 50, *ω*_1_ = 100, *ω*_2_ = 299, *P*_*ref*_ = 0.05.

#### 2.7.2 Functional network

When constructing the functional network, first a zero‐phase‐shift filter was used to extract the characteristic frequency band, and the LFP signal channels were used as nodes of the network to calculate the SL matrix. Then the threshold was selected to binarize the SL coefficient matrix. The value in the SL matrix greater than the threshold was set as 1, indicating a functional connection between the two nodes; the value less than the threshold was set as 0, indicating no active connection between the two nodes. To describe and measure the connectivity changes in functional networks, the clustering coefficient *C* and global efficiency *E* of the network were calculated. The clustering coefficient *C* mainly reflects the degree of closeness between nodes and adjacent nodes in the network. The larger the value of *C*, the denser the connections between nodes. The global efficiency *E* mainly reflects the global transmission capability of the network. The larger the value of *E*, the higher the information transmission efficiency between nodes in the network. The formulas for the clustering coefficient and the global efficiency are as follows:

(8) $$C=\frac{1}{n}\sum_{i=1}^{n}\frac{2e}{k_{i}\left(k_{i}-1\right)}$$

where *k*_*i*_ is the number of nodes adjacent to node *i*, *e*_*i*_ is the actual number of edges connected to node *i*, and *n* is the number of nodes in the network. 0 ≤ *C* ≤ 1, *C* = 1 means the network is fully bonded, and *C* = 0 means that all the nodes in the network are isolated nodes, and there are no connected edges.

(9) $$E=\frac{1}{n\left(n-1\right)}\sum_{1\leq i\leq j\leq n}d_{\mathrm{ij}}^{-1}$$

where *d*_*ij*_ is the shortest path length between nodes *i* and *j* and *n* is the number of nodes in the network.

### 2.8 Histology and electrode registration

**FIGURE 2:**
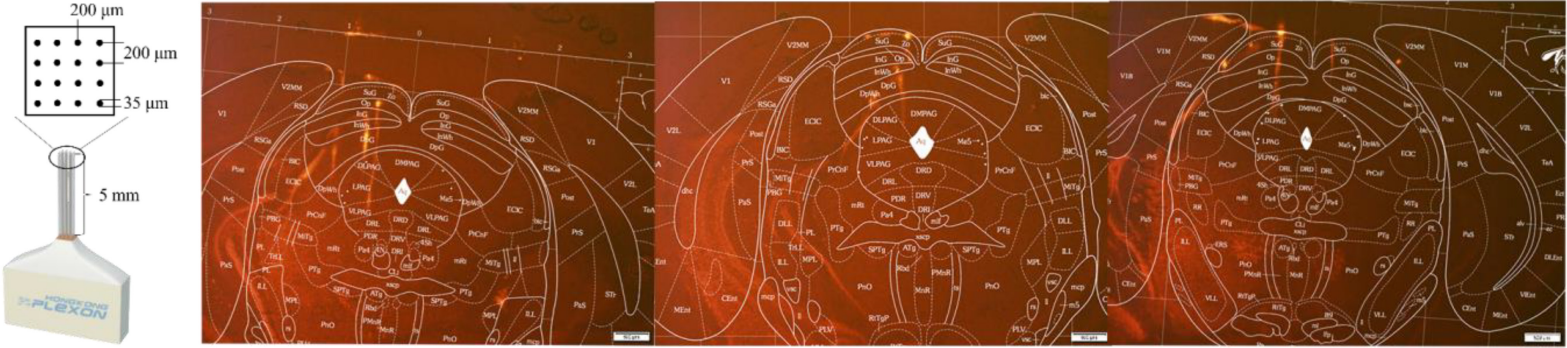
The brain section (coronal, 40 μm thick) shows the recorded area from an experiment: the electrode tracks are marked with DiI.

Animals were perfused with phosphate‐buffered saline and then fixed using 4% paraformaldehyde (PFA) solution. The brains were harvested and placed in PFA (4%) for 48 h; they were sectioned into 40‐μm slices along the coronal plane using a freezing microtome (Leica CM1950, Germany). The sections were examined under a research‐grade whole slide scanning system (Olympus VS200, Japan) to confirm the placement of the electrode marks (DiI fluorescence) within the dPAG (Figure 2).

### 2.9 Statistics

Two‐way analysis of variance (ANOVA) and Tukey's multiple comparisons were used to analyze the changes in frequency band activity and network connectivity.^35^ The significance level was set to 5%, and *p < 0.05, **p < 0.01, ***p < 0.001, and ****p < 0.0001 were considered significant. Data are represented as mean ± SD.

## 3 RESULTS

Neural data of dPAG nuclei in three mice under different innate defensive behaviors were collected. According to De Franceschi et al. and Procacci et al,^11^, ^36^ flight behavior is the mouse quickly returns to the safe area with a speed greater than 40 cm/s; freezing behavior is cessation of all movements (except breathing‐related activities) for at least 0.5 s. After screening, 244 trials, comprising 128 trials for flight behavior and 116 trials for freeze behavior, with obvious defensive responses were obtained. First, the behavioral responses of mice to fear visual stimuli were analyzed; then, the encoding characteristics under the two innate defensive behaviors were analyzed from the perspective of spike firing and LFP oscillations; finally, we constructed a functional network based on the characteristic frequency bands and analyzed the topological property changes in the network. Data analysis was performed using MATLAB 2020b, and statistical analysis was performed using GraphPad 8.

### 3.1 Behavioral responses to visual stimuli

**FIGURE 3:**
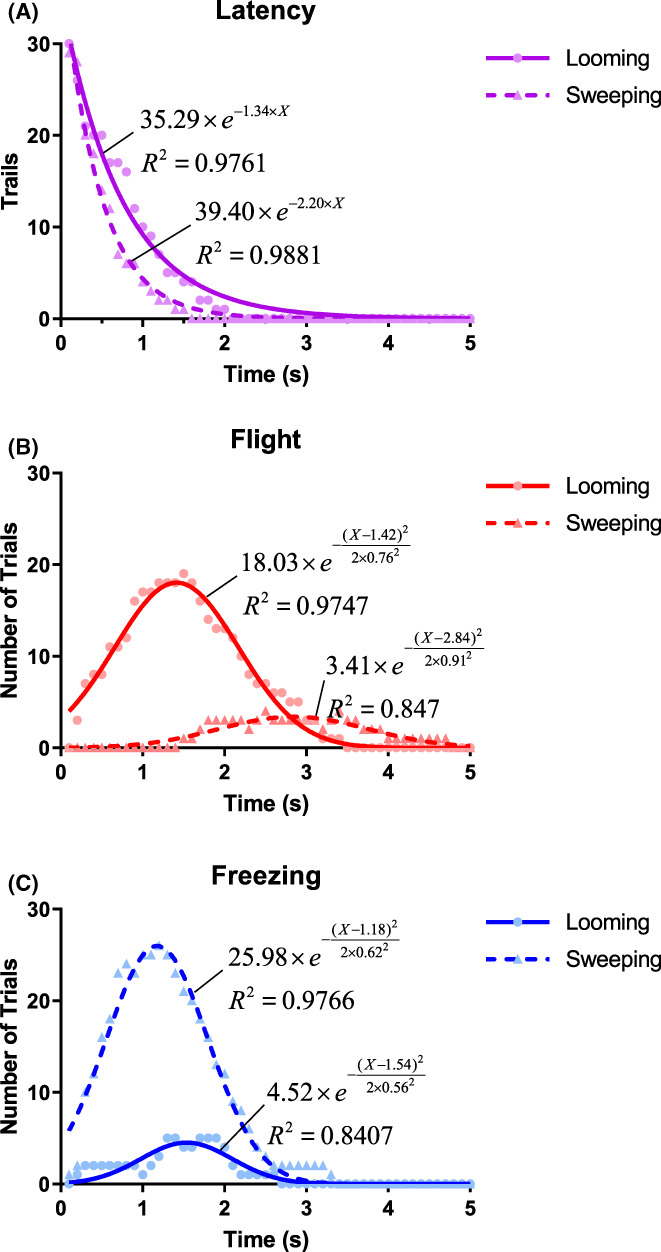
Innate defensive behavioral responses to visual stimuli in mice. (A) Latency distribution of flight/freezing behavior under two kinds of visual stimuli (latency period is defined as the period between the onset of the stimulus and the time before the mouse perceives the visual stimulus and begins to exhibit defensive behavior) (fitting: one‐phase decay, see Section 2.4 for details). (B) Distribution of flight behavior under two kinds of visual stimuli (fitting: Gaussian distribution, see Section 2.4 for details). (C) Distribution of freezing behavior under two kinds of visual stimuli (fitting: Gaussian distribution, see Section 2.4 for details).

The video recordings of partial trials under looming (*n* = 30) and sweeping (*n* = 30) visual stimuli were selected to analyze the behavioral responses of mice. Taking the start time of visual stimulation as 0 and the width of the time window bin as 100 ms, the distribution of the different behavioral states of mice for each bin within 0–5 s was counted, and the fitting curves are shown in Figure 3. The results revealed that the rate constant *K* (*p* < 0.05, looming: 1.34 ± 0.10, sweeping: 2.20 ± 0.12) and *Half‐life* (*p* < 0.05, looming: 0.52 ± 0.04, sweeping: 0.32 ± 0.02) of latency between two visual stimuli showed differences. The *Amplitude* of flight behavior was significantly higher in looming stimuli than in sweeping stimuli (*p* < 0.05, looming: 18.03 ± 0.75, sweeping: 3.41 ± 0.32). In comparison, the *Amplitude* of freezing behavior in sweeping stimuli was higher than that in looming stimuli (*p* < 0.05, sweeping: 25.98 ± 1.11, looming: 4.52 ± 0.57), indicating that looming mainly induces flight behavior and sweeping mainly induces freezing behavior. On the contrary, the time distribution center of maximum probability of freezing behavior caused by sweeping stimulus was smaller than that of flight behavior caused by looming stimulation (*p* < 0.05, freezing: 1.18 ± 0.03 [sweeping] vs. flight: 1.42 ± 0.04 [Looming]), suggesting that the occurrence time of induced freezing behavior is less than that of flight time.

### 3.2 Coding characteristics of spikes under two defensive behaviors

**FIGURE 4:**
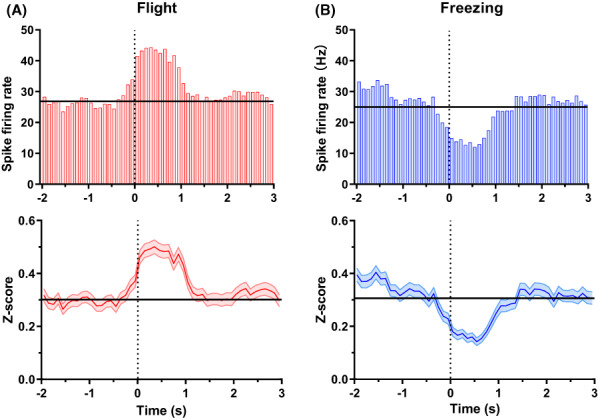
Units' activity in dPAG (dorsal periaqueductal gray) under two defensive behaviors. (A) (top) PSTHs (peri‐stimulus time histograms) of flight‐related units under flight behavior and (bottom) the normalized average firing rate of flight‐related units under flight behavior. (B) (top) PSTHs of freezing‐related units under freezing behavior and (bottom) the normalized average firing rate of freezing‐related units under freezing behavior. The vertical line represents the start of defensive behavior, and the horizontal line represents the baseline.

Taking the start time of defensive behavior as 0, the neural activity of the two defensive behaviors was analyzed, and it was found that the spike firing rate under the flight behavior showed an overall increasing trend. In contrast, a decreasing trend of spike firing rate under freezing behavior was observed. The unit was defined as a flight‐related unit if its firing rate increased by at least 1 Hz in a 1‐s time window after the onset of the defensive behavior compared to the baseline (1 s before the onset of the defensive behavior). Similarly, the unit was defined as freezing related if its firing rate decreased by at least 1 Hz at the same condition. The peri‐stimulus time histograms and the *Z*‐score of the two kinds of units are shown in Figure 4. It can be observed that the neural activities under the two defensive behaviors show different firing patterns. In addition, before the defense behavior occurs, the trend of neural activity has already begun. It appears that after the accumulation of neural firing to a certain threshold, the animal's defensive behavior is triggered.

**FIGURE 5:**
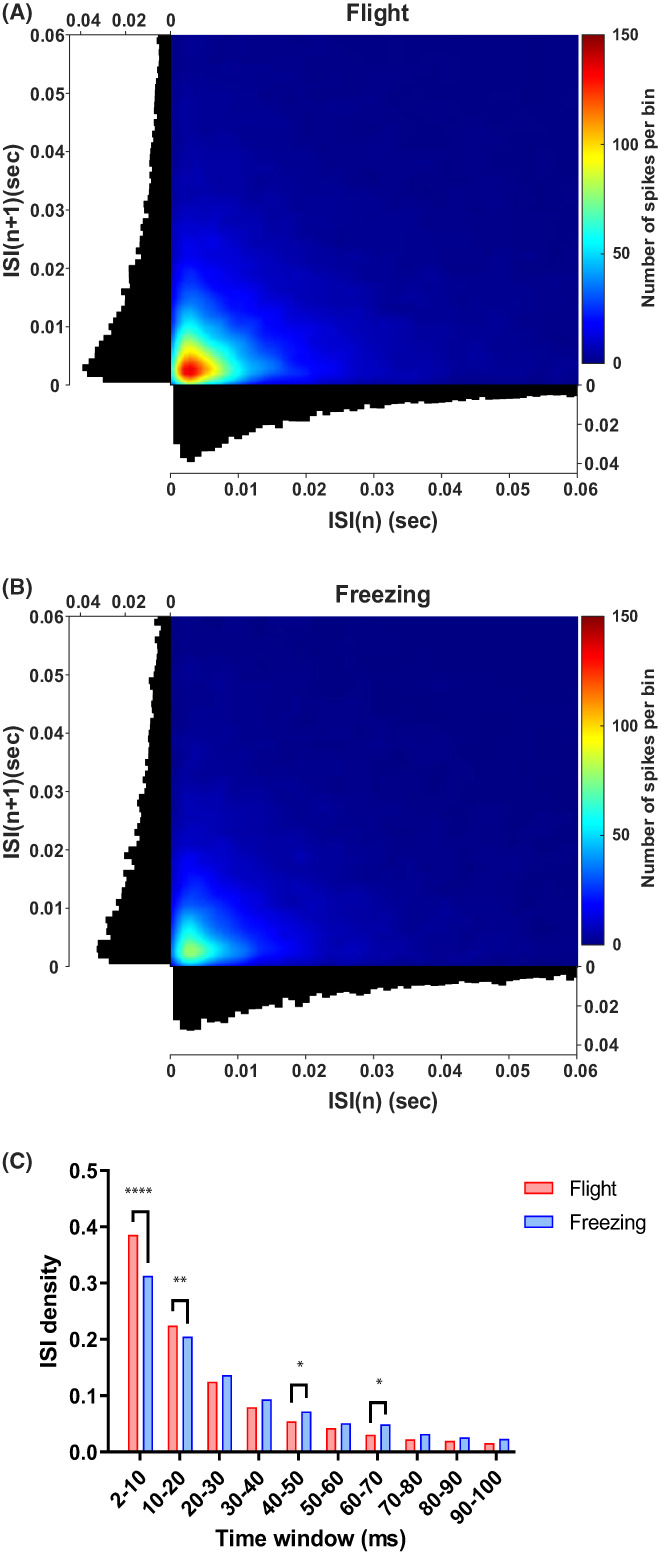
Comparison of spike firing intervals under two defensive behaviors. (A) ISI (inter‐spike interval) density distribution under flight behavior. (B) ISI density distribution under freezing behavior. (C) ISI density distribution comparison between two defensive behaviors. Two‐way ANOVA (analysis of variance) and multiple comparisons were used to test for differences; *p < 0.05, **p < 0.01, and ****p < 0.0001.

Units' activities in the 0–1 s period were intercepted to analyze the distribution of the inter‐spike interval (ISI) in the 2–100 ms time window under the two defensive behaviors (Figure 5). ISI density and the number of spike firings in each time window (bin = 1 ms) under the flight and freezing behaviors are shown in Figure 5(A,B), respectively. And the peak of the ISI density under the flight behavior (0.039) was higher than that under the freezing behavior (0.0325). Then, the ISI density distributions under the two defensive behaviors were counted with the sliding window of bin = 10 ms (Figure 5C). The difference between the two defensive behaviors was most significant at 2–10 ms (*p* < 0.0001), and additionally at 10–20 ms (*p* = 0.0038), 40–50 ms (*p* = 0.0176), and 60–70 ms (*p* = 0.0105), differences were observed. These results suggested that the frequency and intensity of spike firing may be essential features of dPAG encoding different innate defensive behaviors.

### 3.3 Frequency oscillations of LFP under two defensive behaviors

**FIGURE 6:**
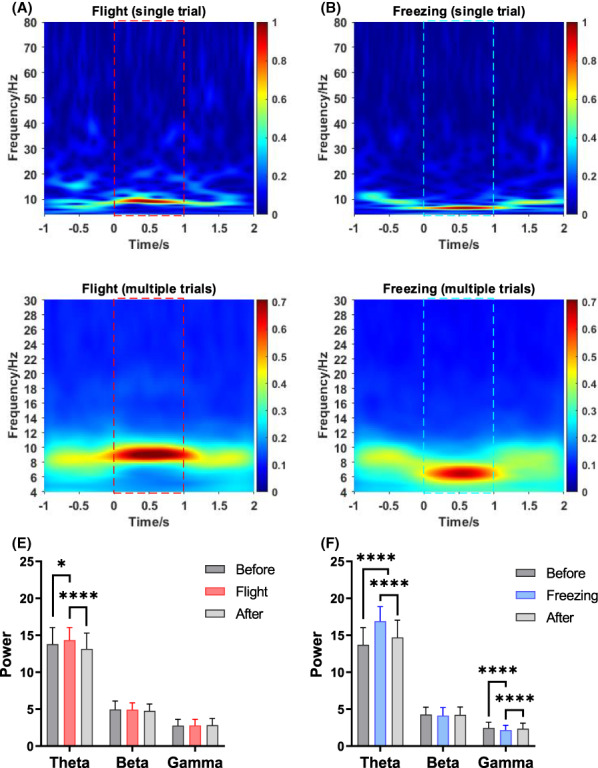
Band activity under two innate defensive behaviors. (A) Band activity under flight behavior (single trial). (B) Band activity under freezing behavior (single trial). (C) Band activity under flight behavior (averaged over multiple trials). (D) Band activity under freezing behavior (averaged over multiple trials). (E) Power variation in different frequency bands under flight behavior. (F) Power variation in different frequency bands under freezing behavior. The red dashed line box is the flight period, and the blue dashed line box is the freezing period. Two‐way ANOVA (analysis of variance) and multiple comparisons were used to compare differences; *p < 0.05, and ****p < 0.0001.

The time–frequency analysis results revealed that the dPAG nuclei exhibited obvious frequency band oscillations when the two innate defense behaviors were generated (Figure 6). Of these, the activity frequency was mainly concentrated at 8–10 Hz under the flight behavior, whereas the activity frequency was primarily concentrated at 6–8 Hz under the freezing behavior. Then the power of theta (4–12 Hz), beta (12–30 Hz), and gamma (30–80 Hz) bands was calculated. It was found that the power of the theta band was significantly enhanced during the innate defense than before and after the defense behaviors. In addition, the gamma band power decreased during freezing compared with the period before and after freezing.

### 3.4 Connectivity changes in functional networks under two defensive behaviors

**FIGURE 7:**
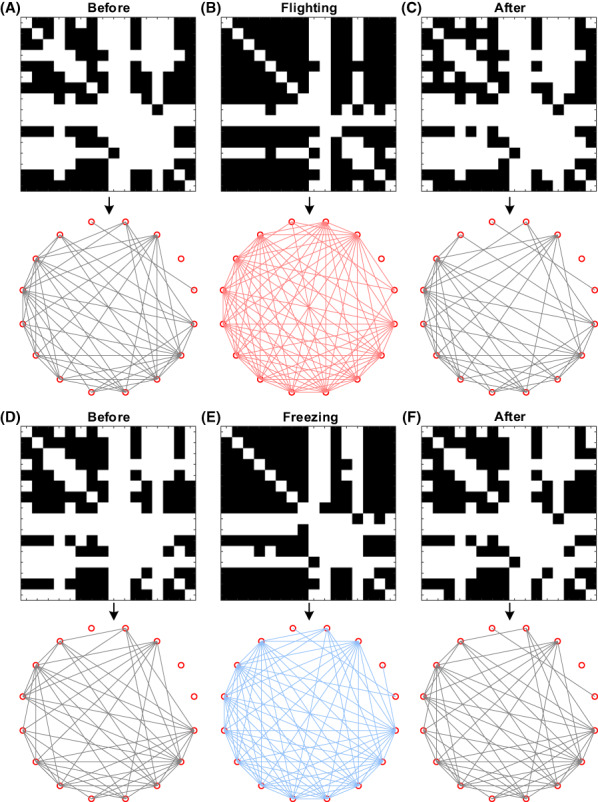
Functional network changes in dPAG (dorsal periaqueductal gray) nuclei under flight and freezing behaviors. (A) Network connection before the flight. (B) Network connection during the flight. (C) Network connection after the flight. (D) Network connection before freezing. (E) Network connection during freezing. (F) Network connection after freezing. In each group of figures, the upper figure represents the binarized adjacency matrix, and the lower figure represents the network connection.

Because the frequency band activities under the two defensive behaviors are mainly concentrated in the theta frequency band, the brain functional network was further constructed based on the theta frequency band, and the topological characteristics were analyzed. The SL coefficient matrices of the three time periods (before, during, and after the defense, the data length of each period was 1 s) in each trial were calculated separately, and the SL matrices of the corresponding periods in all trials were averaged. Based on the mean of the coefficient matrices before and after the defensive behavior, the binarization threshold was set to 0.5409, and the functional network connection is shown in Figure 7. Figure 7A–C shows the network connection changes in three stages of flight behavior, and Figure 7D–F shows the dynamic evolutions during the period of freezing. The results showed that the connectivity of functional network under flight or freezing was significantly higher than that before and after the defense, indicating that there was high synchronization among the LFP signal channels, and most neurons exhibited consistent functionality.

**FIGURE 8:**
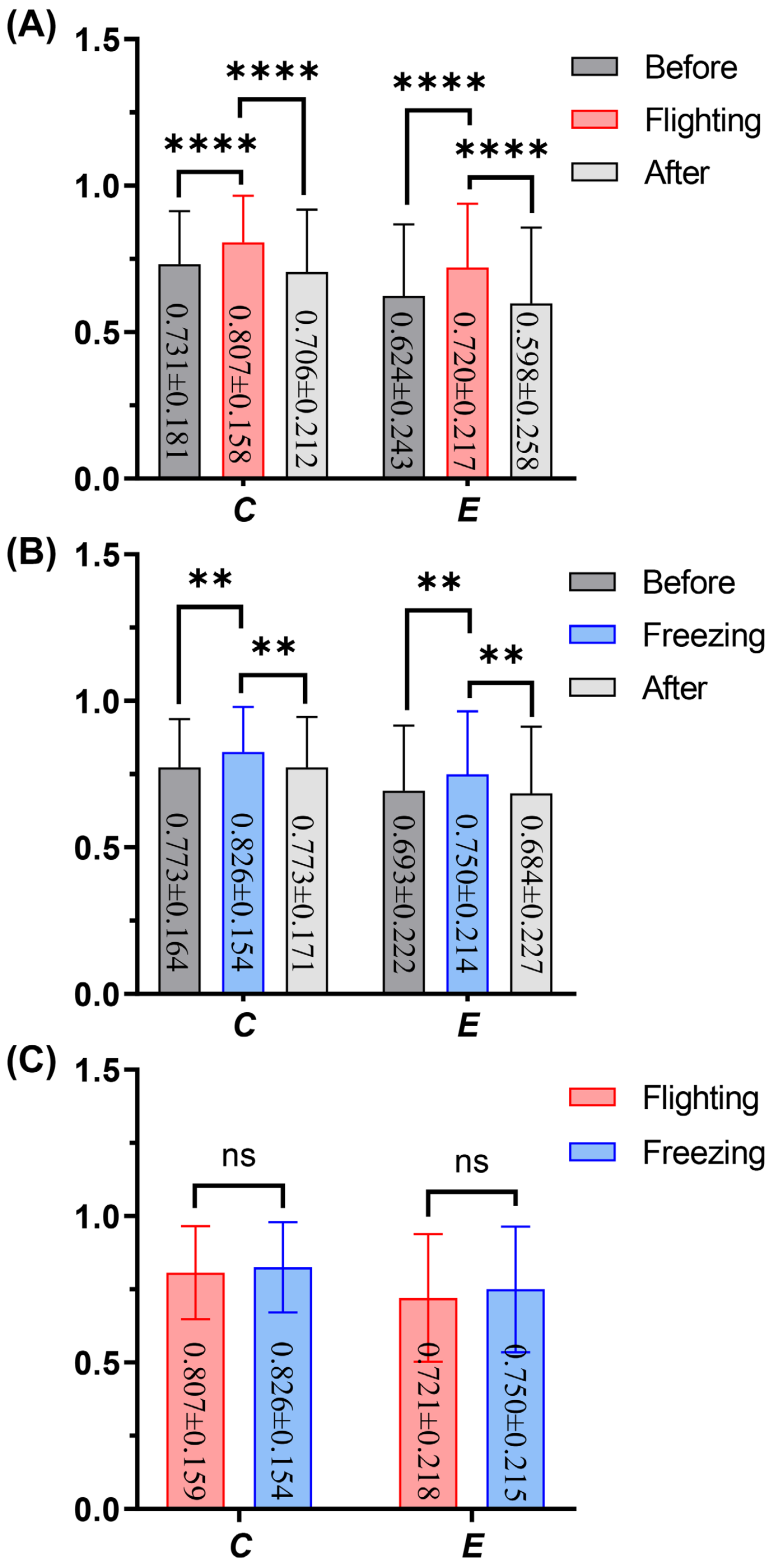
Changes in network topology characteristics under two defensive behaviors. (A) Feature changes when flight behavior occurs. (B) Feature changes when freeze behavior occurs. (C) The contrast in network features between flight and freezing. Two‐way ANOVA (analysis of variance) and multiple comparisons were used for the analysis of differences between groups; **p < 0.01, ****p < 0.0001, and the data are expressed as mean ± SD.

Similarly, the changes in network topology characteristics under the two defensive behaviors showed (Figure 8) that the clustering coefficient *C* and global efficiency *E* during flight were significantly higher than those before and after the flight (*p* < 0.0001), and similarly, the two features (*C* and *E*) during freezing were also higher than those before and after freezing (*p* < 0.01). However, there was no difference in network characteristics between flight and freezing behaviors. These results indicated that high synchronization of the neuronal activities in the dPAG nuclei occurred during the two defensive behaviors, and the connection density and information transmission efficiency among neurons in the specific brain regions significantly improved.

## 4 DISCUSSION

This study adopted looming and sweeping visual stimuli to induce flight and freeze defense behaviors in mice. The neural activity of dPAG nuclei was recorded and analyzed under two defensive behaviors. It was found that neurons in dPAG nuclei exhibit distinct coding characteristics between flight and freezing behaviors. The spike firing frequency and firing interval were significantly different between the two defensive behaviors, and the theta band of the LFP signal oscillated under both defense behaviors. Still, the activity frequencies of the two were different. In addition, there was a high synchronization of neural activity during the defense process. These coding features may be the key factors in the choice of defensive behavior of dPAG nuclei.

Looming is an imminent strong stimulus with a short time delay that can elicit a rapid escape response. Simultaneously, sweeping is a relatively weak stimulus that is more likely to induce a freezing response with a longer delay. The behaviors evoked by the two stimuli used in this paper are divided into strong and weak. Looming can induce stronger and quicker responses, whereas sweeping induces more weak responses to measure the least cost. The fear behavioral responses caused by looming and sweeping are different. The firing of neurons has already started before the behavioral response. While flight behavior tends to be a subconscious instinctual response through a series of neural computations, freezing behavior represents more complex neural computations that make predictive behavioral decisions.

The behavioral responses of mice to visual stimuli show that mice are more inclined to flight during an approaching threat. In contrast, before cruising predators, mice are more prone to freezing to reduce the probability of detection.^11^ In addition, the center distribution time of freezing behavior (1.18 s, induced by sweeping stimulus) is earlier than that of flight behavior (1.42 s, induced by looming stimulus), which may be related to the regulatory mechanism of defensive behavior.^37^ Freezing behaviors consume less energy and show susceptibility, whereas flight behaviors consume more power and require high‐frequency neural activity, showing late onset.

Previous findings have indicated that stimulation of neurons in dPAG resulted in increased flight and freezing behaviors,^26^ whereas dPAG destruction blocked animals' defensive responses to predators.^27^ Similarly, our results suggested that significant neural activation in dPAG nuclei was observed under both innate defensive behaviors, in which flight behavior may be directly driven by the medial superior colliculus‐dPAG,^38^ whereas freezing behavior may be mediated by the superior colliculus‐pulvinar‐lateral amygdala‐PAG neural circuit.^14^

We align the neural data by the onset of defensive behaviors and find that dPAG nuclei exhibit different coding characteristics between the two defensive behaviors. Neural activity was more intense under flight behavior than freezing behavior, associated with significantly higher neural firing rates during flight behavior.^26^, ^39^ Another study found that more neurons of dPAG exhibited negative modulation in freezing behavior than in flight behavior.^40^ And about 0.5 s before the defensive behavior occurs, the neural activity begins to show differences; when the neural activity accumulates to a certain threshold, different types of defensive behaviors are triggered. Neural activity during the defense process exhibited high synchronization, suggesting that neurons in the dPAG nuclei focused on encoding defense strategies.

There are a few studies on the molecular mechanism of transient firing in the nervous system, mainly focusing on the late expression regulation induced by neurotransmitter firing.^41^ However, the pathway or molecular biology is still unclear, and the characterization of related molecules and proteins is still incomplete. Further interventions from the spatiotemporal transcriptome are needed to obtain better results.

## AUTHOR CONTRIBUTIONS

All authors meet the requirements for authorship. Denghui Liu and Shouhao Li wrote the manuscript, Denghui Liu and Shouhao Li performed the experiments, and Liqing Ren managed the animals. Xiaoyuan Li and Zhenlong Wang guided and revised the contents of the first draft of the manuscript. All authors read and approved the final manuscript.

## CONFLICT OF INTEREST

The authors declare that they have no conflicts of interest.
